# Supplementary material for: Association between Sleep Timing, Being Overweight and Meal and Snack Consumption in Children and Adolescents in Southern Brazil
Source: Int J Environ Res Public Health. 2023 Sep 21;20(18):6791. doi: 10.3390/ijerph20186791 (PMC10531239; doi:10.3390/ijerph20186791)
Supplement: Supplementary file 1 [file ijerph-20-06791-s001.zip › ijerph-2492744-supplementary.pdf]

Figure S1. Flow chart of the study design

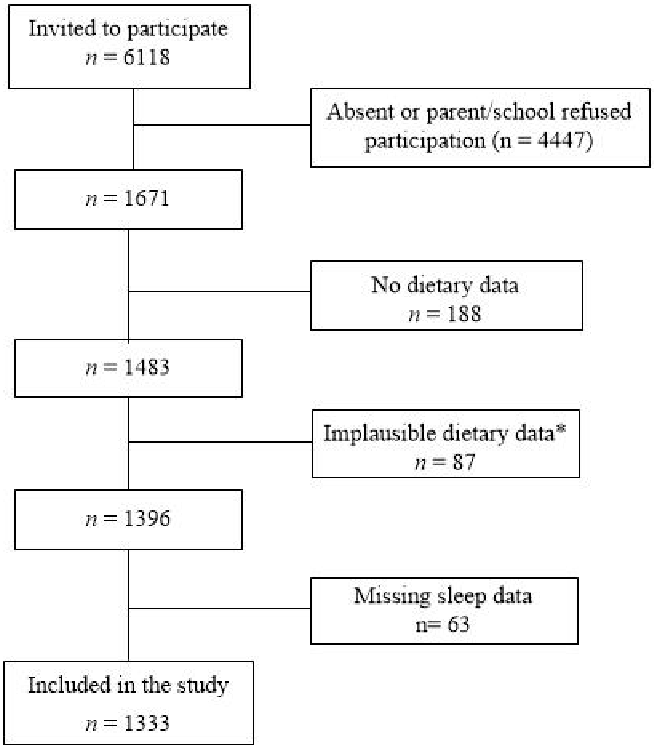

*Children and adolescents who reported less than three food items per day or consumption of a number of items greater than the mean + 3 standard deviations.

Table S1. Description of sleep variables according to the midpoint of sleep. Florianopolis, Brazil, 2018/2019.

**Sleep variables**	**Early**<br>**n=460**	**Intermediate**<br>**n=434**	**Late**<br>**n=439**
	median (p25; p75)	median (p25; p75)	median (p25; p75)
**Sleep duration (h)**			
Total	**9.64 (9.08; 10.29)** [a]	**9.64 (9.11; 10.43)** [c]	**10.14 (9.29; 11.00)** [a,c]
Weekday	**9.50 (8.75; 10.00)** [a]	**9.50 (8.50; 10.50)** [c]	**10.00 (9.00; 11.00)** [a,c]
Weekend	10.00 (9.50; 11.00)	10.50 (10.00; 11.00)	10.50 (9.50; 11.00)
**Bedtime (local time)**			
Weekday	**21:30 (21:00; 22:00)** [a,b]	**22:00 (21:40; 22:35)** [b,c]	**23:00 (22:00; 23:30)** [a,c]
Weekend	**22:00 (22:00; 22:30)** [a,b]	**23:00 (23:00; 23:30)** [b,c]	**0:00 (23:35; 1:00)** [a,c]
**Wake-up time (local time)**			
Weekday	**6:40 (6:30; 7:00)** [a,b]	**7.00 (6:30; 8:30)** [b,c]	**9:00 (7:00; 10:00)** [a,c]
Weekend	**8:00 (8:00; 9:00)** [a,b]	**9:30 (9:00; 10:00)** [b,c]	**10:30 (10:00; 11:00)** [a,c]
**MSFsc**	**3:01 (2:38; 3:17)** [a,b]	**3:35 (3:26; 4:11)** [b,c]	**5:05 (4:38; 5:41)** [a,c]

Bold font and superscripts indicate: Kruskal-Wallis test, **a** that the first and third tertile are significantly different at 5% level; **b** that the first and second tertile are significantly different at 5% level; **c** that the second and third tertiles are significantly different at 5% level.

Table S2. Associations between the midpoint of sleep and mid-afternoon snack, lunch, and dinner. Florianopolis, Brazil, 2018/2019.

	**Lunch**				**Mid-afternoon snack**				**Dinner**			
	**Crude OR (95% CI)**	**p**	**Adjusted[a] OR (95% CI)**	**p**	**Crude OR (95% CI)**	**p**	**Adjusted[a] OR (95% CI)**	**p**	**Crude OR (95% CI)**	**p**	**Adjusted[a] OR (95% CI)**	**p**
**Early**	1.21(0.70;2.09)	0.451	1.18(0.60;2.33)	0.590	0.54(0.25;1.17)	0.104	0.50(0.22;1.15)	0.093	0.91(0.59;1.43)	0.656	0.95(0.54;1.67)	0.838
**Intermediate**	1	-			1				1	-		
**Late**	2.24(0.56;9.06)	0.223	2.68(0.57;12.67)	0.184	0.57(0.33;1.00)	0.050	0.47(0.20;1.09)	0.074	1.02(0.67;1.56)	0.912	1.25(0.89;1.76)	0.167
**Gender**												
Male	1	-	1	-	1	-	1	-	1	-	1	-
Female	0.94(0.43;2.08)	0.869	0.95(0.45;2.04)	0.890	**1.74(1.17;2.58)**	**0.011**	**1.57(1.00;2.45)**	**0.049**	0.78(0.43;1.41)	0.368	0.84(0.41;1.72)	0.593
**Age**												
7-10 years	1	-	1	-	**1**	**-**	**1**	**-**	1	-	1	-
11-14 years	1.71(0.64;4.52)	0.245	2.00(0.81;4.98)	0.119	0.92(0.51;1.67)	0.761	1.02(0.59;1.78)	0.931	1.36(0.61;3.00)	0.406	1.35(0.64;2.83)	0.386
**Screen use**												
never	1	-	1	-	**1**	**-**	**1**	**-**	1	-	1	-
once	1.20(0.22;6.60)	0.810	0.96(0.22;4.30)	0.958	1.19(0.72;1.96)	0.451	1.10(0.58;2.10)	0.746	0.91(0.61;1.37)	0.627	0.84(0.48;1.48)	0.509
twice	1.02(0.15;6.97)	0.980	1.02(0.13;8.11)	0.987	1.04(0.45;2.43)	0.911	1.02(0.42;2.46)	0.970	0.61(0.29;1.28)	0.168	0.62(0.35;1.11)	0.098
three times or more	0.56(0.16;1.93)	0.319	0.43(0.14;1.36)	0.131	1.18(0.69;2.01)	0.506	1.19(0.70;2.04)	0.469	1.45(0.82;2.58)	0.175	1.30(0.69;2.45)	0.370
**Physical activity**												
0-2 times	1	-	1	-	**1**	**-**	**1**	**-**	1	-	1	-
3-4 times	1.17(0.38;3.57)	0.760	1.22(0.38;3.90)	0.713	1.04(0.62;1.73)	0.880	0.96(0.62;1.49)	0.834	1.60(0.94;2.71)	0.076	1.43(0.74;2.76)	0.247
>=5 times	6.30(0.60;66.39)	0.111	7.48(0.57;97.85)	0.110	1.74(1.06;2.87)	0.033	1.56(1.10;2.21)	0.018	5.35(1.35;21.26)	0.022	4.78(1.25;18.20)	0.027
**Type of school**												
Public	1	-	1	-	**1**	**-**	**1**	**-**	1	-	1	-
Private	2.44(1.22;4.89)	0.018	2.18(0.77;6.17)	0.123	1.23(0.95;1.59)	0.107	1.33(0.82;2.16)	0.213	1.61(0.95;2.74)	0.073	1.23(0.57;2.82)	0.586
**Maternal education, years of schooling**												
0-8	1	-	1	-	**1**	**-**	**1**	**-**	1	-	1	-
9-11	2.37(1.03;5.43)	0.043	2.19(1.07;4.50)	0.036	1.85(0.85;4.04)	0.107	1.62(0.88;2.96)	0.107	0.70(0.48;1.01)	0.054	0.66(0.52;0.84)	0.004
≥12	3.50(1.37;8.90)	0.014	2.40(0.52;11.22)	0.230	1.23(0.78;1.92)	0.329	0.86(0.39;1.90)	0.669	1.25(0.79;1.98)	0.297	1.04(0.54;2.01)	0.901
**Day of the food intake report**												
Weekend	1	-	1	-	**1**	**-**	**1**	**-**	1	-	1	-
Weekday	0.48(0.09;2.59)	0.347	0.25(0.04;1.43)	0.106	1.98(0.95;4.13)	0.063	2.02(0.99;4.13)	0.053	1.28(0.68;2;38)	0.399	0.92(0.42;2.02)	0.824
**Weight status**												
Non-overweight	1	-	1	-	**1**	**-**	**1**	**-**	1	-	1	-
Overweight	0.85(0.41;1.73)	0.609	0.96(0.47;1.97)	0.907	0.44(0.30;0.68)	0.002	0.44(0.28;0.69)	0.003	0.84(0.36;1.91)	0.635	0.87(0.35;2.15)	0.731
**Total sleep duration (h)**	0.85 (0.47;1.57)	0.572	0.86(0.48;1.52)	0.560	1.20(0.95;1.53)	0.113	1.27(1.01;1.59)	0.039	0.84 (0.56;1.26)	0.361	0.88(0.61;1.27)	0.456

[a]Adjusted by gender, age, screen use, physical activity, type of school, weight status, maternal education, day of food intake report, and total sleep duration.
Bold values denote statistical significance at the $p < 0.05$ level.
